# New Theory in Regard to Caries of the Teeth

**Published:** 1898-10

**Authors:** 


### NEW THEORY IN REGARD TO CARIES OF THE TEETH.

At the meeting of the California State Dental Association, held at San José in June, 1898, Dr. A. C. Hart, professor of Clinical Dentistry in the College of Physicians and Surgeons of San Francisco, propounded the following theory in regard to dental caries:

He takes the ground that decay is a natural force, acting through media, the chief of which is water on all material bodies. That it is not bacteria, acids, alkalies, salts, alcohol, and oils that cause or prevent decay, but that their action is dependent on water. To bacteria he would ascribe the cause of the fever present in diseases like typhoid, scarlet, yellow, and other fevers that owe their origin to the growth of bacteria. He showed the enormous power bacteria have for consuming water, and ascribed the emaciated condition present in consumption due to the power of abstracting water from the tissues on the part of bacteria. He believed there was some truth at least in the practice as followed by the ancients in refusing a fever patient all the water he wanted. He would combat the action of bacteria by keeping the water protected in the tissues from the solvent action of bacteria. This, he claims, is the manner of action of many of our most powerful antiseptics and germicides. He says,—

"We speak of persons being immune or susceptible to bacterial growth. Immunity, I believe, is the result of the cells becoming so specialized as to be able to place the water of combination insoluble or inaccessible to bacteria, and *vice versa.*

"Oil poured on water will form a film that retards evaporation and growth of bacteria. Bacteria will not grow on tissue covered with any of the essential oils, like oil of cinnamon, because the oil protects the water, or has a greater affinity for the water than have the bacteria. So it is by protecting the water in tissues by treating them with solutions that have a greater affinity for the water in the cell than have the bacteria that our chief antiseptics and germicides owe their power.

"Disease generally makes its appearance through a change in the water, which is ninety per cent. of the body weight. The more stable we can keep the water in the body the greater its power of resisting disease.

"Now for practical application: let us see if we can place the water in the teeth insoluble to bacteria. We know that decay ofttimes becomes checked and the enamel and dentine immune to bacterial growth. I believe this immunity is due to the dentine and enamel having attracted compounds that have a greater affinity for the water in the tooth than have the bacteria, or because the tooth has taken up substances insoluble to the action of bacteria, as is seen in the mouths of users of tobacco.

"I have tried for the past three years to harden enamel and dentine against the action of bacteria, and have been uniformly successful in making the tooth take up substances like formaldehyde, nitrate of silver, sulphate of copper, chloride of zinc, and other substances of a like nature that have proved themselves to be powerful antiseptics and germicides. I believe their power is directly owing to their ability to place the water in the enamel and dentine so as to be insoluble or inaccessible to bacteria. I have treated with success cases where the teeth were covered with white, chalky spots, literally melting away before the action of bacteria, without removing the decay or filling.

"Last year I advocated formalin or formaldehyde as an agent possessed with great power in hardening tooth-substance against the action of bacteria. After another year's experience I can report nothing but success. I believe it is a step in advance when we as a profession recognize and use agents that prevent the action of bacteria on the teeth. We cannot prevent their growth in the mouth, but we can use the means in our hands to stop decay. By so doing we will clear the field of those vultures that prey on the people, and through gilded and glaring signs of 'painless extraction,' ruin their teeth and shorten their lives. But the people must be educated to value their teeth. That they are soft, chalky, and decay because of bacteria. That it is possible to so harden them that they will resist the solvent action of bacteria."

